# The Interactions and Release Kinetics of Sodium Hyaluronate Implemented in Nonionic and Anionic Polymeric Hydrogels, Studied by Immunoenzymatic ELISA Test

**DOI:** 10.3390/pharmaceutics14010058

**Published:** 2021-12-27

**Authors:** Dorota Wójcik-Pastuszka, Aleksandra Skrzypczyk, Witold Musiał

**Affiliations:** Department of Physical Chemistry and Biophysics, Faculty of Pharmacy, Wroclaw Medical University, ul. Borowska 211A, 55-556 Wroclaw, Poland; dorota.wojcik-pastuszka@umw.edu.pl (D.W.-P.); skrzypczyk.aleksandra94@gmail.com (A.S.)

**Keywords:** hyaluronan, viscosity, kinetics, drug release, wound therapy

## Abstract

Hyaluronan is a natural polymer that was introduced to wound therapy. Formulations based on synthetic polymers such as methylcellulose (MC) and polyacrylic acid (PA) containing hyaluronan (HA) were proposed for the development of prospective wound-healing preparations. The formulations of different carrier concentrations containing a fixed amount of HA were prepared, and their viscosity was measured. The HA release was evaluated by employing the apparatus paddle over a disc. The hydrogels were introduced to the donor chamber, and HA was released to the pH = 7.4 buffer. The amount of HA released was obtained using the ELISA test. The release was analyzed on the basis of different kinetic models: zero-, first-, and second-order kinetics, as well as Higuchi and Korsmeyer–Peppas equations. The release rate constants and the half release time were calculated from these equations. According to the value of the coefficient of the determination, the best model describing the observed process was selected. The comparison between the dissolution profiles was carried out by calculating the difference factor f_1_ and the similarity factor f_2_. The interaction between the hydrogel components was investigated by Fourier-transform infrared spectroscopy (FTIR) and differential scanning calorimetry (DSC) measurements. The study revealed that the zero-order equation best described the release of HA from the formulations studied. The FTIR research and the DSC study showed the intermolecular interaction between HA chains in MC-based compositions, as well as between HA and the synthetic polymer in the PA-based formulations. The study revealed that the formulation with a higher concentration of synthetic polymer may prolong the release of HA. The obtained results indicated that the proposed hydrogels have potential for wound healing and may accelerate skin regeneration.

## 1. Introduction

Hyaluronic acid is a natural polymer that exists as a polyanion in all living organisms. It is non-epimerized linear glycosaminoglycan. The chain consists of repeated d-glucuronic acid and d-*N*-acetyl-glucosamine connected through beta-1,4 and beta-1,3 glycosidic bonds [1]. This biomolecule is present in the body as a salt form—hyaluronan (HA). It occurs in the extracellular matrix of the skin, joints, eye, and many other tissues and organs. A high concentration of HA was also found in the lung, kidney, brain, and muscle tissues [2]. The hyaluronan moiety can create internal hydrogen bonds, as well as interact with a solvent. HA possesses good solubility in water and has a high level of the viscosity even when the amount of biopolymer is very low [3]. In a dilute solution, HA may exist as a separate molecule; however, with the increase in the biopolymer concentration, three-dimensional web structures are observed. A concentration of HA greater than 0.1 mg/mL with a molecular weight of about 5.0 MDa is capable of forming an internal net that ensures lubricious and very hydrophilic properties. Depending on the length of the chain and the mass of the molecule, there are high-, medium-, and low-molecular-weight forms [4,5], The low-molecular-weight form of this polymer increases inflammation, self-destruction processes, and angiogenesis. High-molecular-weight HA has an anti-inflammatory function and protects tissues against apoptosis and phagocytosis. HA is the basic structural element of the extracellular matrix and plays an important role in the wound-healing process. This is due to its hydrophilic, rheological, and viscoelastic properties. The interaction of HA with blood components creates a scaffold on which the granulation tissue is formed and then a scar. The binding of HA with water causes swelling and enables inflow of nutrients into the wound. It has been revealed that low-molecular-weight HA fragments increase the expression of type I collagen formed during the fibrosis and scarring process [6,7]. HA is used in pharmacy for the preparation of modified release drug forms and preparations used in a targeted therapy. It is also used to obtain hydrogels that are carriers of protein drugs. HA can be conjugated with a drug or used to produce microcapsules transporting the drug to the appropriate area of the body. Modified derivatives of HA are used in the transport of nucleotide drugs because their carboxyl groups form a crosslinked DNA-binding hydrogel [4,5,8].

Hydrogels are a very comfortable preparations employed for skin and mucous membrane applications. Hydrogels based on cellulose derivatives and polyacrylic acid are very stable. They can be used on damaged skin, they are easy to remove from the skin surface, and they provide a feeling of cooling and hydration. It was observed that the incorporation of selected active substances into the hydrogel does not cause sedimentation [9,10]. Hydrogels are sensitive to a variety of physical and chemical conditions including temperature, electric or magnetic fields, light, pressure, sound, pH, solvent composition, and ionic strength [11]. Polyacrylic acid (PA) introduced into water dissociates, and the obtained solution becomes acidic. The neutralization of the ionized carboxyl groups leads to the formation of a gel due to the electrostatic repulsion between the charged polymer chains. The viscosity of preparations based on PA is low at pH values less than 3 or greater than 12 [12]. Methylcellulose (MC) is a cellulose derivative, in which the glucan hydroxyl groups are substituted with CH_3_O groups. The degree of substitution and the chain length affect its physical properties such as the viscosity and the degree of the solubility. MC swells under the influence of water, forming transparent gels with a neutral pH [13]. Such hydrogels have potential applications in drug delivery formulations.

The study conducted by Catazano et al. [14] revealed that the formulation of alginate and hyaluronan has a beneficial effect in the treatment of wounds. Thönes et al. [15] proposed a hydrogel composed of HA and collagen containing sulfated HA for wound therapy. They found that compositions that may bind and release HA are reservoirs that induce the prolonged release of the active substance.

The aim of this work was to evaluate a new polymer-based composition containing HA for the development of a prospective wound-healing preparation, via an investigation of the interaction between the carrier and HA, and a determination of the kinetics of the release of HA from the obtained formulations.

## 2. Materials and Methods

### 2.1. Materials

Sodium hyaluronate (HA) and methylcellulose (MC) were purchased in Sigma-Aldrich (St. Louis, MO, USA). HA was obtained from rooster comb and was expected to have a molecular weight of 1–4 million. Polyacrylic acid (PA) was supplied from Lubrizol (Brumach, Belgium). Sodium hydroxide and potassium dihydrogen phosphate anhydrous were provided by Chempur (Piekary Śląskie, Poland). The phosphate buffer solution pH = 7.4 was prepared according to European Pharmacopoeia 10.5 [16].

### 2.2. Preparation of Hydrogels

The appropriate amount of the carrier was mixed with water to obtain the right concentration of the polymer in the final formulation. In the case of using PA, the pH of the hydrogel was adjusted to ca. 8.0 by adding 10% NaOH solution. The value of the pH of the hydrogel was controlled by a multifunction meter used as a pH meter (CX-601, ELMETRON, Zabrze, Poland) connected with the electrode (ERH-12-6, ELMETRON, Zabrze, Poland). Then, 0.125 g of HA was mixed with water and added to the hydrogel. The final formulation was left for 24 h at the temperature of 6 °C to remove bubbles of air. The composition of the prepared formulations is shown in Table 1.

### 2.3. Viscosity Test

The prepared formulations were placed in a water bath (PR-1, Labo Play, Bytom, Poland) until the desired temperature was reached. The viscosity of the hydrogels was measured at the temperature of 37 °C by employing a rotational viscometer (Brookfield DV2T, Middleboro, MA, USA). The rotation speed of the spindle was fixed at 200 rpm, and spindle no. 5 or 6 was mainly used. The measurement parameters such as the rotation speed and spindle no. were chosen to obtain scale coverage between 60% and 90%. Each measurement was performed three times in order to calculate the average value of the viscosity of the hydrogel (η) together with the standard deviation (SD).

### 2.4. Release Study of HA from Hydrogels

According to European Pharmacopoeia [16], the release investigation of HA from hydrogels F1–F3 and F7–F9 was carried out by employing a five-paddle apparatus over a disc. The dissolution test was performed at a temperature of 37 °C with a paddle rotation speed of 50 rpm. The appropriate amount of the hydrogel was put onto a disc covered with a semipermeable membrane and placed in the acceptor fluid. The drug was released in 1 L of the phosphate buffer solution, pH = 7.4. Then, 150 μL samples were taken at defined time intervals and replenished with fresh acceptor fluid. Each test was performed six times. The collected samples were stored in a refrigerator at a temperature of 6 °C. The amount of HA released was determined using the ELISA test. This test has already been used in previous studies to determine HA levels [17]. The known amounts of HA were introduced to 16 wells to obtain the calibration curve. Then, 100 μL samples were placed in 80 wells. The absorbance of the formed HA–ELISA complex was measured at 405 nm using a UV/Vis microplate spectrophotometer (Thermo Fisher Scientific, Multiscan Go, Waltham, MA, USA). According to the obtained absorbance–concentration relationship of HA, a standard curve was generated. It allowed determining the amount of HA released from formulations. The obtained dependence of changes in the HA concentration in the acceptor fluid in time was analyzed using zero-, first-, and second- order kinetics, as well as Higuchi and Korsmeyer–Peppas models [18]. The equations used in the kinetic study were presented in our previous study [19].

### 2.5. Mathematical Comparison Equations

The mathematical models allowing the comparison between the drug release profiles proposed by Moore and Flanner, and accepted by FDA were employed to study the variability in the release pattern of HA from hydrogels [20,21]. Using the equations presented below, the difference factor f_1_ and the similarity factor f_2_ were calculated.

(1) $$f_{1}=\frac{\sum_{t=1}^{n}\left|R_{t}-T_{t}\right|}{\sum_{t=1}^{n}R_{t}}\times 100,$$

(2) $$f_{2}=50\times \log\left\{{\left[1+\frac{\sum_{t=1}^{n}{\left(R_{t}-{\;T}_{t}\right)}^{2}}{n}\right]}^{-0.5}\times 100\right\},$$

where n is the number of dissolution timepoints, and R_t_, and T_t_ are the released value of the reference and the test batch at time t, respectively [21].

### 2.6. FTIR Study

The hydrogels F1–F12 were dried at a temperature of 6 °C and grated to a powder before the FTIR study was carried out. The spectra of pure ingredients, formulations F1–F12, and the physical mixtures consisting of the corresponding components were collected, employing an FTIR spectrometer (Thermo Scientific Nicolet iS50, Waltham, MA, USA) coupled to an ATR module (Thermo Scientific Nicolet iS50, Waltham, MA, USA). The measurements were performed in the wavelength range of 500–4000 cm^−1^ at a resolution of 4 cm^−1^. For each spectrum, the average of 32 scans was collected. The obtained spectra were analyzed using a dedicated computer program, OMNIC Spectra 2.0.

### 2.7. DSC Investigation

Dry formulations F1–F12 grated to a powder, their pure components, and physical mixtures of corresponding ingredients were studied using differential scanning calorimetry (DSC). The study was carried out by employing a differential scanning calorimeter (DSC 214 Polyma, Netzsch, Selb, Germany). Samples of 3–5 mg were measured in sealed aluminum pans under a nitrogen atmosphere, with a flow rate of 50 mL/min. The thermograms were scanned at a constant heating rate of 5 K/min in the temperature range from −10 to 300 °C.

### 2.8. Statistical Analysis

All calculated physicochemical parameters in the work were presented as the means ± SD. The influence of the carrier on the viscosity of the hydrogels, as well as on the release profiles of HA, was studied employing the one-way analysis of variance model (ANOVA) and Tukey’s HSD test (honestly significant difference) [22]. The Student *t*-test was also used to compare the dissolution profiles of HA from formulations F1–F3, as well as from formulations F7–F9. The minimum significance level was assumed to be 0.05 [18].

## 3. Results and Discussion

### 3.1. Viscosity Test

**Figure 1:**
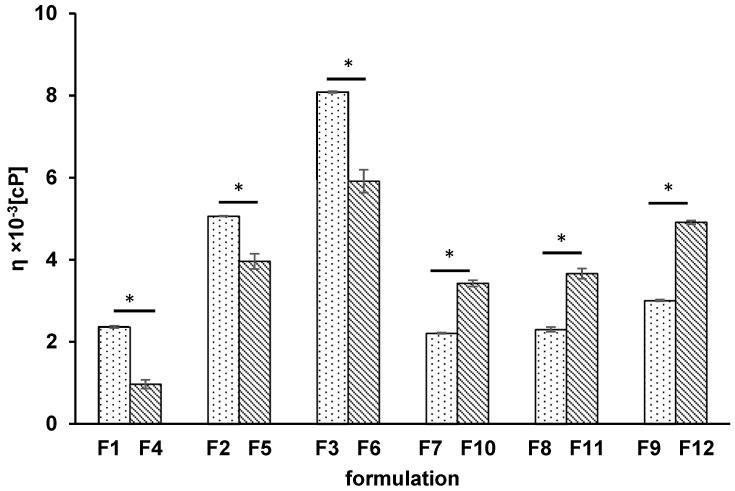
The viscosity of formulations F1–F12 at a temperature of 37 °C; columns with dots reflect formulations with HA, while columns with lines indicate formulations free of HA; asterisks indicate a significant difference at *p* ≤ 0.05 between the formulations with and without HA.

The obtained results from the viscosity study of formulations F1–F12 are presented in Figure 1.

The lowest value of the hydrogel viscosity was observed when the concentration of the synthetic polymer was the lowest, in good agreement with the general rule. The statistical ANOVA of the hydrogel viscosity revealed the differences between the viscosity of formulations F1–F12. The obtained values of the discrepancies between the means are listed in Table 2. It was found that there was no difference in viscosity between F7 and F8 and the corresponding formulations F10 and F11. This result may be explained by the very close concentration of the PA-based hydrogels. In the case of MC-based compositions, differences were noticed between the formulations.

It was revealed that the addition of HA to the hydrogel of MC resulted in an increase in the viscosity of formulations F1–F3 that was in the range from 2.36 ± 0.03 × 10^3^ cP to 8.08 ± 0.02 × 10^3^ cP in comparison to the viscosity of corresponding formulations not containing HA (F4–F6) that was in the viscosity range from 0.97 ± 0.10 × 10^3^ cP to 5.91 ± 0.28 × 10^3^ cP. Consequently, the viscosity of MC-based formulations containing HA was maximally 2.4 times higher than the viscosity of the corresponding formulations free of HA. However, in the case of hydrogels of PA, the effect of the incorporation of HA into hydrogels was different. The viscosity of formulations F7–F9 with HA was from 2.21 ± 0.02 × 10^3^ cP to 3.01 ± 0.02 × 10^3^ cP and was about 0.6 times lower than the viscosity of the corresponding hydrogels of F10–F12 that was from 3.42 ± 0.07 × 10^3^ cP to 4.91 ± 0.04 × 10^3^ cP. This may have been due to the different properties of the synthetic polymers. MC is a nonionic polymer but PA is an anionic polymer, and they interact differently with HA, which is also an anionic polymer.

### 3.2. Kinetics of HA Release

**Figure 2:**
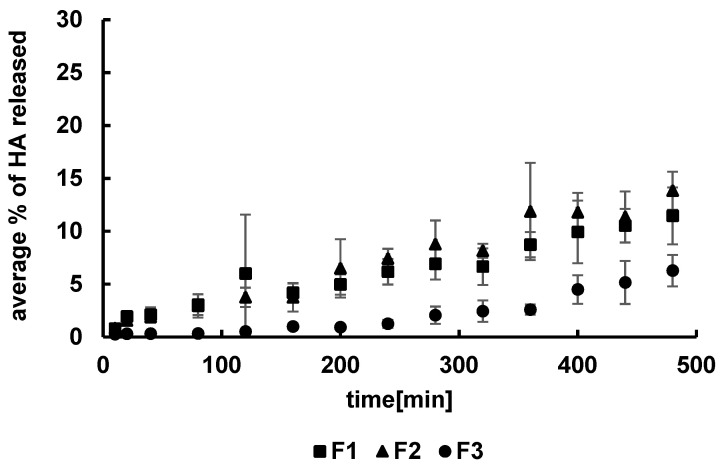
The release profiles of HA from formulations F1–F3.

The release profiles of HA from formulations F1–F3 are presented in Figure 2. The amounts of HA released within 8 h from the MC-based formulations were in the range from 6 ± 1% to 14 ± 2%.

The released HA percentages were 11 ± 3% from F1, 14 ± 2% from F2, and 6 ± 1% from F3. The highest amount of HA was released from formulation F2, although it was very close to the amount of HA released from F1. The lowest amount of HA within 8 h was released from formulation F3. The concentrations of the synthetic polymer in F1 and in F2 were lower than in F3. The results suggest that the concentration of MC influenced the drug release.

**Figure 3:**
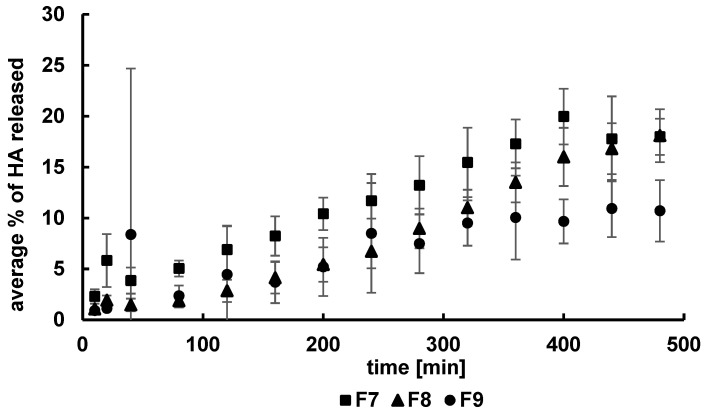
The release profiles of HA from formulations F7–F9.

It may be proposed that MC molecules were a steric hindrance and interfered with the migration of the long HA chains from the carrier to the acceptor fluid, resulting in a reduction in the amount of the natural polymer released. The dissolution curves of HA from formulations F7–F9 are shown in Figure 3. The greatest amount of HA, i.e., 18 ± 3%, was released from the F7 and F8 formulations with the lowest PA concentration. The concentration of HA released from the F9 hydrogel with the highest level of PA was 11 ± 3%. The increase in the PA concentration resulted in a decrease in the amount of the drug released, similar to the case of HA dissolution from the MC hydrogels.

This observation means that the molecules of the PA carrier were also a steric hindrance toward the long chains of HA. The results obtained in the present work were consistent with the outcomes presented by Szcześniak et al. [23] that studied the release of hydrocortisone from PA hydrogels and Liu et al. [24] that tested the dissolution of lidocaine from PA hydrogels. Khan et al. experimentally confirmed [25] a decrease in the amount of the drug released when the polymer concentration increased in the formulation.

Comparing the amounts of HA released from MC and PA compositions, it was interesting to note that the amount of HA released from MC hydrogels was lower than from PA formulations. PA is an anionic polymer, and the release of HA, which is also an anionic polymer, occurs faster than from MC hydrogel, which is a nonionic polymer.

Hydrogels are promising materials for wound treatment. Hassan et al. [26] and Tamer et al. [27] determined that a hydrophilic membrane composed of chitosan and HA is a promising candidate for wound treatment. The lack of steric hindrance and the lack of interaction between the polymers reduce the polymer network deformation and improve the water uptake, as well as enable the absorption of wound exudates. Tamer et al. [28] studied PVA/kaolin composite hydrogels. It was found that the water uptake capabilities of hydrogels facilitate the penetration of cells and promotes the absorption of exudate from the wounds.

**Figure 4:**
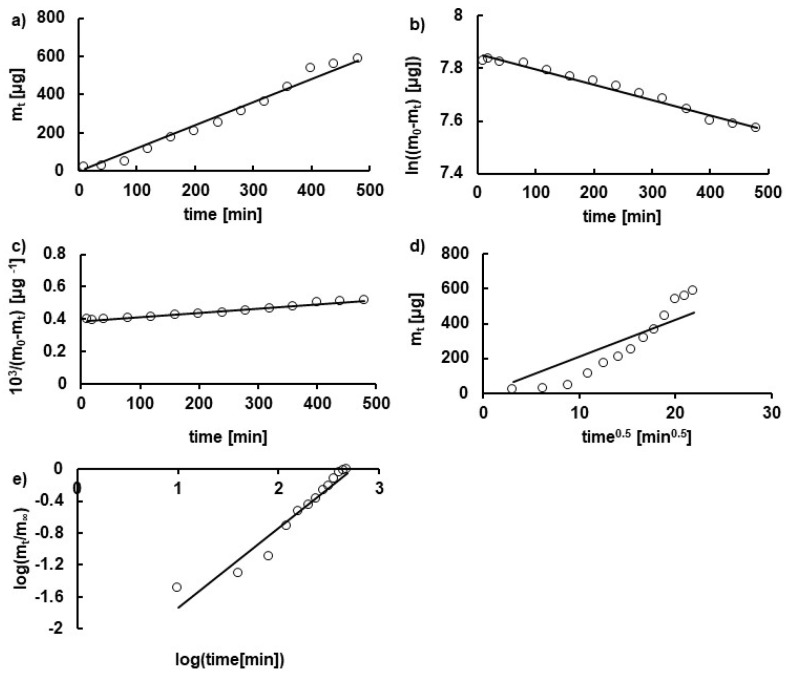
The profiles of HA release from formulation F8 fitted to (**a**) zero-order, (**b**) first-order, (**c**) second-order, (**d**) Higuchi, and (**e**) Korsmeyer–Peppas models.

The dissolution profiles were analyzed employing several kinetic models, and the plots of the experimental points fitted to the theoretical curves are presented in Figure 4. The kinetic parameters such as the release rate constant k, the half release time t_0.5_, and the correlation coefficient *R*^2^ obtained from the analysis are collected in Table 3 and Table 4. It was revealed that the best equations describing the HA release from all formulations studied was the zero-order model. However, the values of correlation coefficients obtained from the model were lower in the case of HA release from formulations F1–F3 than from formulations F7–F9, being in the range from 0.96 ± 0.03 to 0.98 ± 0.02 and from 0.86 ± 0.06 to 0.94 ± 0.03, respectively. It should be mentioned that, in the case of the release of HA from F3, the K–P model was also best fitted. The release rate constants calculated according to the zero-order equation indicated that HA was released in the slowest manner from formulations F3 and F9 containing the highest concentrations of MC and PA, respectively. Additionally, the release rate constants from F3 and F9 were also the lowest using the other models. This was connected to the viscosity of the hydrogels. The increase in viscosity resulted in a decrease in the values of the release rate constants and an increase in the half release time. The same dependency was found by Arora et al. [29] in a study of the dissolution of domperidone from tara gum-based matrix tablets. The results may be explained by the migration of the long HA chains between the carrier molecules that account for a steric barrier and hinder the penetration of the drug into the acceptor fluid. The increase in the viscosity of the hydrogel reflects an increase in the concentration of the matrix polymer and, therefore, greater steric hindrance.

The values of the parameter n obtained from the Korsmeyer–Peppas model were higher than 0.5 in all tests, being in the range of 0.66 ± 0.14–0.86 ± 0.30 when HA was released from hydrogels F1–F3 and in the range of 0.59 ± 0.1–0.93 ± 0.2 when HA was released from F7–F9 formulations. These results indicated that the mass transport followed an anomalous transport [18,30]. They also confirmed the steric hindrance of the free passage of the drug into the acceptor fluid.

### 3.3. The Difference Factor f_1_ and the Similarity Factor f_2_

A comparison between the dissolution curves was carried out, and the difference factor and the similarity factor were calculated. According to FDA recommendations, a difference in the release profile occurs when the value of the f_1_ factor is higher than 15 and the value of the f_2_ factor is below 50. The calculated values of the difference factor f_1_ and the similarity factor f_2_ are listed in Table 5. The obtained values of f_1_ for the release of HA from formulations F1–F3 composed of MC were in the range from 21.2 to 70.4, indicating that the concentration of the synthetic polymer affects the drug release. This was consistent with the results obtained for the HA dissolution from PA hydrogels. The calculated values of the difference factor were in the range from 50.2 to 85.7, highlighting the differences between the drug release profiles from F7–F9.

The analysis of similarity factors revealed that the values of f_2_ obtained from the comparison of HA release profiles from MC hydrogels were in the range from 63.0 to 86.5, highlighting the difference between the dissolution profiles of HA from F1–F3 formulations. The comparison of HA release profiles from F8 and F9 compositions confirmed the discrepancy between formulations. However, the values of f_2_ obtained from the comparison of HA release profiles in F7 and F8, as well as in F7 and F9, were below 50, meaning that there was no difference.

The results obtained from f_1_ and f_2_ analysis were consistent with the results from the release data. A lower concentration of the synthetic polymer favors faster transport of the drug into the acceptor fluid. An increase in the hydrogel concentration or the viscosity hinders the migration of long HA chains, which may encounter steric obstacles from the polymer molecules and reach the acceptor medium more slowly.

### 3.4. Statistical Analysis

The statistical analysis based on the ANOVA method with Tukey’s HSD test revealed that statistically significant differences between release profiles of HA from MC hydrogels were observed in the case of the dissolution of HA from F1–F3 and F2–F3. Differences in the drug release from F1 and F2 were not observed. The same statistical differences between the release profiles of HA from F1–F3 were obtained using the Student *t*-test. The results showed that the concentration of MC may influence the drug release pattern, although the concentration differences of MC in formulations F1 and F2 were not sufficient to notice the discrepancies. No statistically significant differences were obtained when the ANOVA method with Tukey’s HSD test was employed to compare the dissolution profiles of the drug from formulations F7–F9 composed of PA. However, the Student *t*-test indicated the differences between the curves of the release of HA from F7 and F9. The results may suggest that the differences in the concentration of PA in hydrogels F7–F9 were not enough to clearly notice the influence of the polymer concentration on the drug release.

### 3.5. FTIR Study

**Figure 5:**
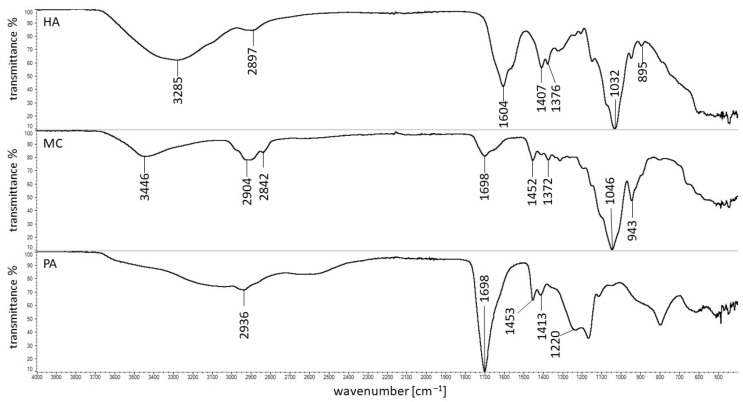
The FTIR spectra of pure HA, MC, and PA.

The FTIR spectra of pure ingredients of formulations F1–F12 are shown in Figure 5. The characteristic maxima of HA were in good correlation with the results obtained by Reddy et al. [31] and Alkrad et al. [32]. The characteristic band of HA at the frequency of 3285 cm^−1^ was assigned to the stretching of the NH group with a combination of C=O, and the maximum at 2897 cm^−1^ indicated the stretching of the C–H group. The sharp peak at 1604 cm^−1^ was assigned to asymmetrical C=O stretching. The signal at 1407 cm^−1^ was related to the symmetrical C–O stretching of the –COO^−^ group. Additionally, the lack of the maximum at 1743 cm^−1^ assigned to the asymmetrical C=O stretching of –COOH indicated the deprotonated form of HA. This was also confirmed by the absence of bands at 1651 cm^−1^ and 1558 cm^−1^, corresponding to amide I and amide II, respectively. They were overlapped by the strong signals at 1604 cm^−1^ and 1407 cm^−1^ [33]. The band at 1376 cm^−1^ was attributed to the deformation of CH, CH_2_, and CH_3_ groups. The strong maximum at 1032 cm^−1^ corresponded to the stretching of the groups C–O–C, C–O, and C–O–H. The peak at 895 cm^−1^ was assigned to the presence of the C–O–C group, as well as the deformation of the carbonyl and hydroxyl groups [31,32].

The peaks of MC presented in Figure 5 were in good correlation with literature data [34,35,36,37]. The maximum at 3446 cm^−1^ was connected with stretching vibration of the –OH group. The bands found at 2904 cm^−1^ and 2842 cm^−1^ corresponded to asymmetric and symmetric C–H stretching vibrations. The bonds observed at 1698 cm^−1^ and 1046 cm^−1^ belonged to C–O and C–O–C groups, respectively. The signals at 1452 cm^−1^ and 1372 cm^−1^ were attributed to the vibration of deformation in the plane of the C–H group. The peak observed at 943 cm^−1^ belonged to the OCH_3_ group. The characteristic band of PA at 2936 cm^−1^ was responsible for the stretching between C–H atoms, and that at 1698 cm^−1^ indicated the presence of the carboxyl group. The maxima at the wavenumbers of 1453 and 1413 cm^−1^ were assigned to the scissors and bending vibrations of CH_2_ and CHCO. The signal at 1220 cm^−1^ was attributed to OH bending from the carboxyl group [38,39,40].

**Figure 6:**
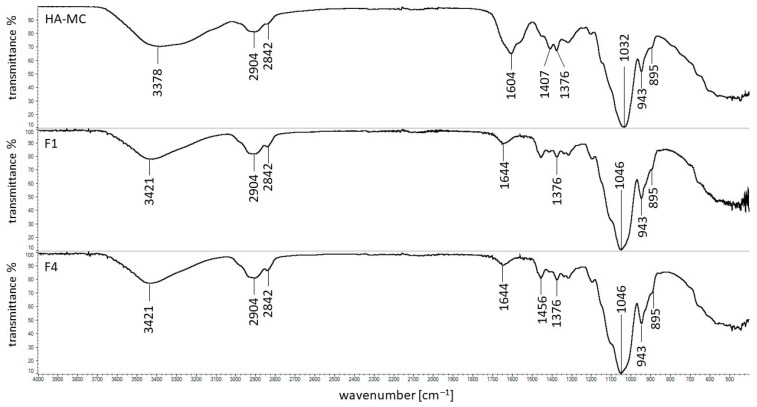
The FTIR spectra of the physical mixture of F1 (HA–MC), formulation F1, and formulation F4.

The spectra of all formulations studied were recorded. As an example, the spectra of formulations F1, F4, and the physical mixture of F1 (HA–MC) are presented in Figure 6.

It was found that all characteristic bands of HA and MC were noticed on the spectrum of the physical mixture. In the case of the spectrum F1, the peak at 1698 cm^−1^ assigned to MC was not present. This signal was also not found on the spectrum of formulation F4, not containing HA. Additionally, the new maximum at 1644 cm^−1^ was observed on F1 and F4 spectra. This may indicate the influence of water. Moreover, the characteristic peak of HA at 1604 cm^−1^ disappeared on the spectrum of the F1 formulation. This may have been due to the overlapping of signals at 1644 cm^−1^ with the maximum at 1604 cm^−1^ or an intramolecular interaction between the carboxyl and amide groups of HA.

**Figure 7:**
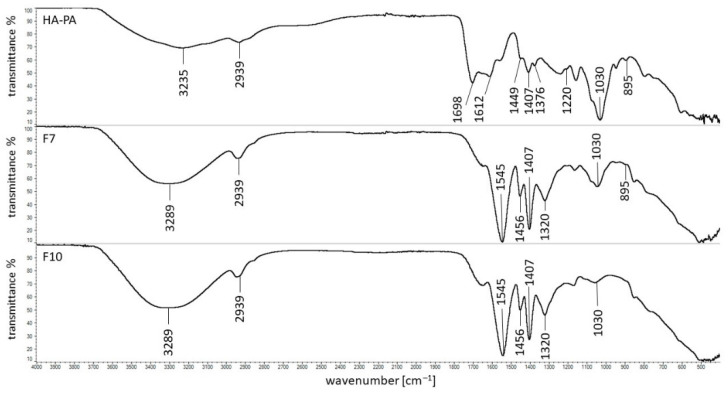
The FTIR spectra of the physical mixture F7 (HA–PA), formulation F7, and formulation F10.

The FTIR spectra of the physical mixture of HA and PA (HA–PA), as well as formulations F7 and F10, are presented in Figure 7.

All characteristic bands of HA and PA were present on the spectrum of the physical mixture F7. However, the peak of PA at 1698 cm^−1^ was shifted to 1545 cm^−1^ on the spectra F7 and F10. Moreover, the additional band at 1644 cm^−1^ was noticed on both spectra. This may suggest the interaction of the polymer with a water molecule because formulation F7 consisted only of PA and water, similar to spectra of F1 and F4 formulations in which MC was used as the carrier. On the spectrum of F7, the characteristic maximum of HA at 1604 cm^−1^ was not observed. This may be explained by the overlap of the bands at 1644 cm^−1^ and 1545 cm^−1^ on the signal at 1604 cm^−1^. The lack of the maximum at 1604 cm^−1^ may also suggest the interaction between HA and PA. A bond between the carboxyl group of PA and the –NH group of the drug—verapamil hydrochloride—was revealed by Elkheshena et al. [41].

The differences between the spectra of MC and PA powders and the corresponding spectra of these polymers in the form of a gel allow us to conclude that water interacts with these compounds. An intermolecular interaction of the carboxyl and amide groups of HA in MC-based compositions, as well as an interaction between HA and PA, may also occur.

The FTIR results corresponded well with the release data. The intermolecular interaction between HA molecules in MC hydrogels resulted in a low amount of HA release that was in the range from 6 ± 1% to 12 ± 3%. However, the bond formation between HA and PA may explain the HA release within 8 h in the range from 11 ± 3% to 18 ± 3%.

### 3.6. DSC Investigation

**Figure 8:**
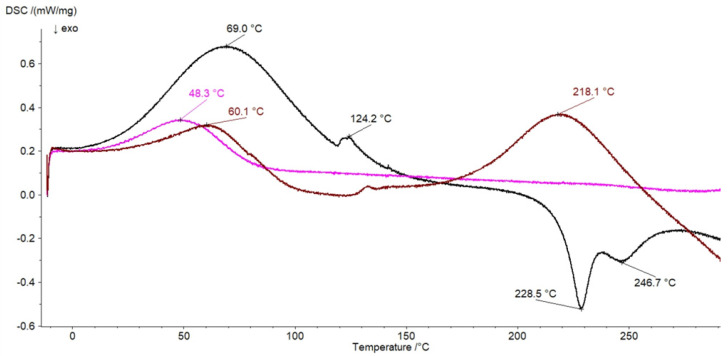
The thermograms of HA (black), MC (pink), and PA (brown).

The thermograms of pure ingredients of the formulations studied are shown in Figure 8.

**Figure 9:**
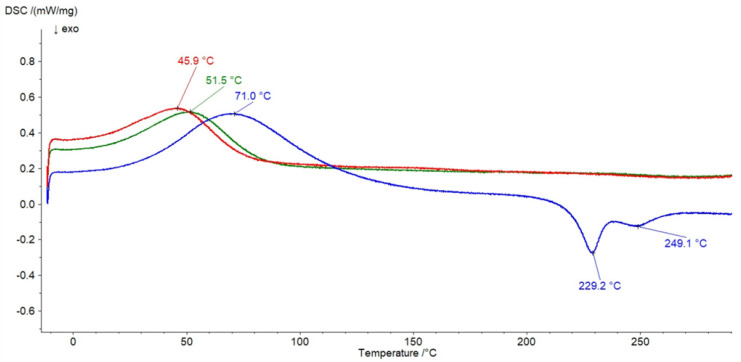
The thermograms of formulation F1 (red), formulation F4 (green), and physical mixture of HA and MC (blue).

Two endotherms of HA connected with loss of water were located at 69 °C and 124.2 °C. Two exotherms indicating the degradation of the polysaccharide were at 228.5 °C and 246.7 °C. The results correspond well with the data obtained by Viletti et al. [42], Benesova et al. [43], Sadeghi-Ghadi et al. [44], and Sgorla et al. [45]. The thermal decomposition curve of MC presented in Figure 8 showed one endothermic maximum at 48.3 °C that can be assigned to the polymer dehydration, which corresponded well with the thermal profile of MC obtained by Niemczyk-Soczyńska at el. [46]. The thermal profiles of F1, F4, and the physical mixture of HA and MC are shown in Figure 9. The analysis of the curve of the physical mixture of HA and MC revealed that all thermal signals coming from HA and MC were present. On the thermogram of the F4 formulation, the maximum at 45.9 °C coming from MC was noticed. The thermal analysis of the F1 preparation showed the presence of the endotherm of MC at 51.5 °C. However, the maxima coming from HA at 124.2 °C, 228.5 °C, and 246.7 °C were not identified on the thermogram of F1. The same observations were obtained from the analysis of the thermograms of F2 and F3 formulations containing MC and HA but with different concentrations of the synthetic polymer. This may be connected to the intermolecular interaction between HA chains. The possible formation of hydrogen bonds among –COOH, –OH, –NHCOCH_3_ groups in the hydrogel of HA was proposed by Luan et al. [33].

**Figure 10:**
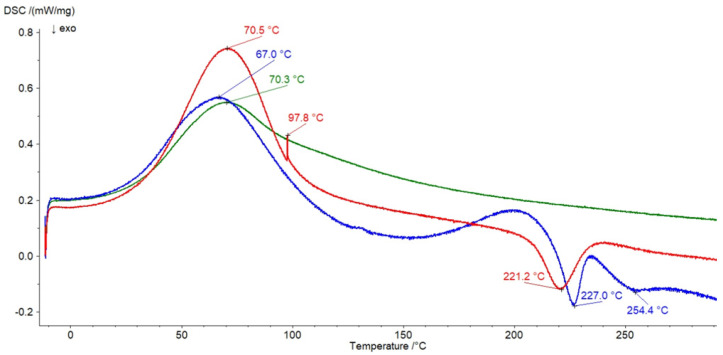
The thermograms of formulation F7 (red), formulation F10 (green), and the physical mixture of HA and PA (blue).

The thermal profile of PA shown in Figure 8 consisted of an endotherm at 60.1 °C indicating the evaporation of water and a second endotherm at 218.1 °C that may be assigned to the melting process. Similar peaks were observed by Neira et al. [39]. The thermograms of formulations F7 and F10, as well as the physical mixture of HA and PA (HA–PA), are presented in Figure 10. All maxima of HA and PA were found on the thermogram of their physical mixture. However, the lack of an endotherm at 218.1 °C belonging to PA on the curve of F10 was noticed. This formulation consisted only of PA and water, meaning that an interaction between the synthetic polymer and the water molecule occurred. On the thermal profile of the F7 formulation, the maximum at 218.1 °C also disappeared, confirming the interaction between PA and water. Additionally, the exotherm at 246.7 °C assigned to HA was not observed on the thermogram of F7, suggesting an interaction between HA and PA. The presence of the exotherm of HA at 221.2 °C on the F7 thermogram may have been the result of its interaction with PA. Binding of HA with the synthetic polymer protects HA against the thermal decomposition. The same results were obtained from the analysis of thermograms of F8 and F9 formulations with the same qualitative composition as F7. The study results were consistent with the FTIR research and the investigation conducted by Elkheshen et al. [41]. It was postulated that a bond between the carbonyl group of PA and the –NH– group of the drug was formed.

## 4. Conclusions

This study showed that the incorporation of HA solution into the MC hydrogel increased the viscosity of the formulation, although the addition of HA to the PA hydrogel decreased the viscosity of the composition. The dissolution study showed that the released amount of HA depends on the viscosity of the hydrogel. The released amount of HA decreased with the increase in the viscosity of the carrier. The kinetic study indicated that the release process was best described by the zero-order equation. The dissolution of HA was faster when the concentration of the synthetic polymer was lower, confirming that the MC and PA molecules were a hindrance in the transport of HA to the acceptor medium. Moreover, the FTIR and DSC investigation revealed that HA may interact with the synthetic molecules. The obtained results suggest that the MC and PA hydrogels may be beneficial carriers in wound therapies that employ controlled release of hyaluronan. The conducted research confirmed that the proposed hydrogels can be a reservoir of HA, and an appropriately selected concentration of the synthetic polymer enables achievement of the sustained release effect.

## Figures and Tables

**Table 1:** The composition of formulations F1–F12.

Formulation	F1	F2	F3	F4	F5	F6	F7	F8	F9	F10	F11	F12
MC (g)	5.00	6.25	7.50	5.00	6.25	7.50	-	-	-	-	-	-
PA (g)	-	-	-	-	-	-	0.56	0.66	0.75	0.56	0.66	0.75
HA (g)	0.125	0.125	0.125	-	-	-	0.125	0.125	0.125	-	-	-
H_2_O (g)	250	250	250	250	250	250	250	250	250	250	250	250

**Table 2:** The differences between means obtained from Tukey’s test; the value of HSD was 0.364.

	F2	F3	F4	F5	F6	F7	F8	F9	F10	F11	F12
F1	2.702	5.722	1.396	1.598	3.552	0.151	0.060	0.645	1.061	1.300	2.548
F2		3.020	4.098	1.103	0.850	2.853	2.762	2.057	1.641	1.402	0.153
F3			7.118	4.123	2.170	5.873	5.782	5.077	4.661	4.422	3.173
F4				2.995	4.948	1.245	1.336	2.041	2.457	2.696	3.945
F5					1.953	1.749	1.659	0.953	0.537	0.299	0.950
F6						3.703	3.612	2.907	2.491	2.252	1.003
F7							0.091	0.796	1.212	1.451	2.699
F8								0.705	1.121	1.360	2.609
F9									0.416	0.655	1.903
F10										0.239	1.487
F11											1.249

**Table 3:** The kinetic parameters of HA release from formulations F1–F3.

Kinetic Model	Kinetic Parameters	F1	F2	F3
Z-O	k_0_ (μg·min^−1^)	0.60 ± 0.09	0.74 ± 0.10	0.24 ± 0.53
	t_0.5_ (min)	2089 ± 295	1734 ± 233	5327 ± 1224
	*R* ^2^	0.93 ± 0.07	0.94 ± 0.03	0.86 ± 0.06
F-O	k_1_ × 10^4^ (min^−1^)	2.2 ± 0.7	2.9 ± 0.8	1.2 ± 0.4
	t_0.5_ (min)	3316 ± 1137	2395 ± 683	5833 ± 2012
	*R* ^2^	0.78 ± 0.23	0.81 ± 0.10	0.75 ± 0.10
S-O	k_2_ × 10^8^ (μg^−1^·min^−1^)	9.5 ± 3.3	0.12 ± 0.40	5.2 ± 1.7
	t_0.5_ (min)	4494 ± 1598	3192 ±946	8171 ± 2872
	*R* ^2^	0.77 ± 0.24	0.80 ± 0.10	0.75 ± 0.10
H	k_H_ (μg·min^−1/2^)	11.1 ± 1.8	13.3 ± 2.2	4.1 ± 1.3
	t_0.5_ (min)	13,538 ± 3797	9368 ± 3104	101,671 ± 67,553
	*R* ^2^	0.72 ± 0.18	0.73 ± 0.07	0.51 ± 0.08
K–P	k_K–P_× 10^2^ (min^−n^)	2.1 ± 1.0	1.0 ± 0.5	0.3 ± 0.2
	t_0.5_ (min)	203 ± 344	242 ± 471	506 ± 2428
	*R* ^2^	0.87 ± 0.08	0.87 ± 0.08	0.74 ± 0.08
	*n*	0.66 ± 0.14	0.74 ± 0.16	0.86 ± 0.30
best fit		Z-O	Z-O	Z-O, K–P

Z-O, zero-order model; F-O, first-order model; S-O, second-order model; H, Higuchi model; K–P, Korsmeyer–Peppas model.

**Table 4:** The kinetic parameters of HA release from formulations F7–F9.

Kinetic Model	Kinetic Parameters	F7	F8	F9
Z-O	k_0_ (μg·min^−1^)	1.2 ± 1.3	1.0 ± 0.08	0.66 ± 0.8
	t_0.5_ (min)	1154 ± 129	1427 ± 117	2125 ± 236
	*R* ^2^	0.96 ± 0.02	0.98 ± 0.01	0.96 ± 0.03
F-O	k_1_ × 10^4^ (min^−1^)	4.1 ± 0.9	4.3 ± 0.5	2.6 ± 0.6
	t_0.5_ (min)	1711 ± 385	1641 ± 211	2863 ± 620
	*R* ^2^	0.87 ± 0.07	0.95 ± 0.02	0.87 ± 0.08
S-O	k_2_ × 10^7^ (μg^−1^·min^−1^)	1.7 ± 0.4	1.7 ± 0.2	1.1 ± 0.2
	t_0.5_ (min)	2198 ± 512	2149 ± 300	3883 ± 865
	*R* ^2^	0.86 ± 0.08	0.95 ± 0.02	0.87 ± 0.09
H	k_H_ (μg·min^−1/2^)	21.6 ± 2.3	17.8 ± 3.2	11.8 ± 1.8
	t_0.5_ (min)	4064 ± 907	6826 ± 2613	14,880 ± 4827
	*R* ^2^	0.84 ± 0.04	0.73 ± 0.07	0.77 ± 0.09
K–P	k_K–P_ × 10^2^ (min^−n^)	3.0 ± 1.3	0.3 ± 0.1	1.1 ± 0.6
	t_0.5_ (min)	150 ± 216	287 ± 549	211 ± 457
	*R* ^2^	0.89 ± 0.09	0.88 ± 0.03	0.87 ± 0.07
	*n*	0.59 ± 0.1	0.93 ± 0.2	0.73 ± 0.2
best fit		Z-O	Z-O	Z-O

**Table 5:** The calculated values of the difference factor f_1_ and the similarity factor f_2_.

	f_1_		f_2_	
	F2	F3	F2	F3
F1	21.2	66.5	86.5	67.8
F2	-	70.4	-	63.0
	F8	F9	F8	F9
F7	58.5	85.7	44.5	37.3
F8	-	50.2	-	61.2
